# Special Issue “Advances in Proteomics in Cancer”

**DOI:** 10.3390/ijms262411969

**Published:** 2025-12-12

**Authors:** Leo Kei Iwai

**Affiliations:** Laboratory of Applied Toxinology, Center of Toxins, Immune-Response and Cell Signaling LETA/CeTICS, Butantan Institute, Av Vital Brasil, 1500, Sao Paulo 05503-900, Brazil; leo.iwai@butantan.gov.br

Over the past decade, the paradigm of molecular oncology has decisively shifted from a primarily gene-centric view to a deeply integrated, multi-omics perspective. While genomics and transcriptomics provide an essential blueprint of malignant potential mutations, copy number variations, and transcriptional programs, it is proteomics that delivers a quantitative and dynamic portrait of the functional effectors that execute cellular processes. The long-held assumption that transcript levels (as measured by RNA-seq) reliably proxy protein abundance has been repeatedly challenged. Numerous studies have confirmed that the correlation between transcriptomics and proteomics is often modest, at best [1,2]. This discordance is largely attributable to the complex, multi-layered regulation at translational and post-translational levels that governs protein synthesis, localization, interaction, and degradation. Therefore, to truly understand the phenotypic state of a cancer cell, its signaling activity, metabolic rewiring, structural integrity, and therapeutic vulnerabilities, a direct, in-depth analysis of the proteome is not merely advantageous but fundamentally necessary.

This imperative has been met by rapid technological progress in mass spectrometry (MS). The maturation of liquid chromatography–tandem mass spectrometry (LC-MS/MS), powered by high-resolution and high-speed analyzers such as the Orbitrap and advanced time-of-flight (TOF) instruments, has transformed the field. What was once a low-throughput endeavor struggling to identify hundreds of proteins is now a robust discipline capable of quantifying many thousands of proteins from complex tumor lysates in a single experiment using Data-Independent Acquisition (DIA) or advanced Data-Dependent Acquisition (DDA) strategies [3]. Moreover, single-cell proteomics has emerged as an important tool for directly measuring functional protein heterogeneity at the level of individual cancer cells [4]. This depth of analysis has moved the field from simple cataloging to systems-level quantitative biology.

Perhaps the most profound insights of the last ten years have emerged from the study of post-translational modifications (PTMs). Cancer is, in many aspects, a disease of aberrant PTMs. Among these, phosphoproteomics stands as the most pervasive and dynamically regulated modification, acting as a principal mechanism by which oncogenic signaling is propagated. Phosphoproteomics, the mapping of kinase-driven signaling networks, has rightfully become a cornerstone of targeted therapy development and resistance studies [5,6]. The centrality of phosphorylation is underscored by the fact that kinase dysregulation is among the most recurrent molecular events in tumorigenesis, shaping proliferation, survival, metabolic rewiring, and immune evasion [7]. Moreover, the transient and reversible nature of phosphorylation provides an unparalleled window into real-time pathway activation, enabling proteomics to capture rapid signal-dependent state changes that cannot be inferred from static genomic or transcriptomic profiles. At the same time, the appreciation of other PTMs has grown immensely. We now recognize that the “PTM code” is far more complex. Glycosylation, for instance, is a hallmark of cancer. Aberrant cell-surface glycans, driven by dysregulated glycosyltransferases, fundamentally alter cell–cell adhesion (e.g., the E-cadherin to N-cadherin switch in epithelial–mesenchymal transition), modulate receptor tyrosine kinase signaling, and facilitate immune evasion by masking antigens or engaging inhibitory lectins [8,9]. Concurrently, ubiquitination, the focus of one article in this Special Issue, has emerged from its traditional view as a simple degradation tag to being recognized as a sophisticated signaling language (involving mono-, poly-, and branched-ubiquitin chains) that controls protein localization, enzymatic activity, and DNA repair, rendering the E3 ligase–substrate axis a prime therapeutic target [10].

This analytical power is fully realized in the context of proteogenomics. This integrative approach leverages patient-specific genomic and transcriptomic data to create customized protein sequence databases. The synergy enables the unambiguous identification of peptides arising from single-nucleotide variants (SNVs), gene fusions, or unannotated splice isoforms, directly linking genomic aberrations to their functional protein-level consequences. The field is actively working toward translation. Enormous effort is now directed at developing robust, multiplexed, targeted-MS assays (such as Parallel/Selective Reaction Monitoring, or PRM/SRM) for validating biomarker candidates, and at tackling the immense analytical challenge of plasma proteomics for non-invasive liquid biopsies, aiming to detect tumor-shed proteins at plasma concentrations as low as the attomolar range [11].

This Special Issue, entitled “Advances in Proteomics in Cancer,” provides a valuable and timely collection of articles that exemplify these cutting-edge themes. It features seven outstanding contributions, two comprehensive reviews, and five original research articles that showcase the breadth of modern cancer proteomics, spanning methodological innovation, PTM-driven signal transduction, biomarker discovery, and novel therapeutic development.

Two comprehensive review articles provide a survey of high-impact areas. Agostini et al. (contribution 1) deliver a detailed review of proteomic investigations into immune checkpoints and their inhibitors. This work is particularly relevant as proteomic methods, including imaging mass cytometry (IMC) and targeted MS, are becoming essential for quantifying checkpoint protein expression, such as PD-L1, understanding the protein–protein interaction networks governing T-cell activation, and identifying proteomic signatures that may predict patient response to immunotherapy. Conversely, Arcos et al. (contribution 2) explore the frontiers of bioprospecting, providing a comprehensive proteomic analysis of scorpion venom. Their review illuminates the venom as a complex biochemical library of bioactive peptides and enzymes, many of which exhibit potent anti-tumor properties, presenting a rich and underexplored source for anticancer drug discovery.

The original research contributions in this issue highlight the diverse applications of advanced proteomic techniques to solve specific, pressing problems in oncology. A central theme is the methodological advancement. Juanes-Velasco et al. (contribution 3) present a rigorous and systematic evaluation of the crucial parameters for immunopeptidome characterization. The optimization of HLA-peptide enrichment and MS analysis is vital for accurately identifying the antigenic peptides presented by tumor cells, a foundational step for the development of personalized cancer vaccines and adoptive T-cell therapies. Addressing a different technical bottleneck, Manteghi et al. (contribution 4) introduce the “Peptosome,” a novel peptide-based transfection tool. Their work demonstrates its high efficiency as a non-viral vector, offering a promising alternative to liposomes for the delivery of genetic material in cancer gene therapy research, thereby bridging the gap between genomic-level intervention and its functional outcome.

Other contributions apply these advanced techniques to decipher the complex biology of cancer. Li et al. (contribution 5) conduct an ambitious multi-omics characterization of E3 ubiquitin ligase regulatory patterns across diverse cancer types. By integrating proteomic data with other omics layers, their study begins to unravel the complex and heterogeneous dysregulation of the ubiquitin-proteasome system, reinforcing its importance as a critical regulator of cellular homeostasis and a high-priority therapeutic target. In a notable example of applied phosphoproteomics, Todorov et al. (contribution 6) perform a quantitative mapping of the signaling networks associated with L1CAM overexpression in high-grade serous ovarian carcinoma. Their findings mechanistically link L1CAM to radioresistance and tumorigenesis, identifying specific downstream signaling nodes that could be targeted to sensitize these highly aggressive tumors to therapy. Finally, Pagano et al. (contribution 7) utilize proteomics to explore tumor cell plasticity in a novel environment: simulated microgravity. Their investigation of pancreatic ductal adenocarcinoma (PDAC) cells reveals significant proteomic and phenotypic alterations and identifies the histone deacetylase (HDAC) inhibitor Trichostatin A (TSA) as a potential agent to restore a more normal cellular phenotype, opening a new, unexpected avenue for therapeutic investigation in this recalcitrant disease.

Taken together, these seven articles highlight the dynamism and critical impact of proteomics in contemporary oncology. They demonstrate a field that has matured from qualitative description to quantitative, functional, and translational science. Looking forward, the next decade promises even more radical transformations. The rapid emergence and refinement of single-cell proteomics technologies (e.g., SCoPE-MS, diaPASEF, and nanoflow proteomics) is poised to finally resolve the profound intratumoral heterogeneity that underpins metastatic dormancy, clonal evolution, and therapeutic resistance, providing insights that bulk tumor analysis inherently obscures. The integration of spatial proteomics will further allow us to map these complex protein networks within the native tissue architecture, revealing the critical interactions between tumor cells and their microenvironment. This exponential increase in data complexity and scale necessitates a parallel evolution in computational biology, where artificial intelligence and machine learning will become indispensable for data interpretation, pattern recognition, and predictive modeling.

The ultimate challenge remains translating these discoveries from the bench to bedside. Bridging a statistically significant proteomic signature with a clinically validated, robust, and cost-effective biomarker assay for patient stratification remains a substantial challenge, requiring not only a multi-disciplinary approach but also global collaboration among multiple research groups. Nonetheless, the insights gained from the research presented in this Special Issue, and from the field at large, are invaluable. They are steadily dismantling the black boxes of cancer biology and paving the way for a new generation of more precise diagnostic tools, rational therapeutic combinations, and the ultimate realization of personalized cancer medicine.
